# Effects of grafting on chemical constituents, toxicological properties, antithrombotic activity, and myocardial infarction protection of styrax secreted from the trunk of *Liquidambar orientalis* Mill

**DOI:** 10.1371/journal.pone.0289894

**Published:** 2024-01-05

**Authors:** Shen Huang, Jiayi Cai, Li Ma, Quanlong Zhang, Yiqi Sun, Qiaoyan Zhang, Luping Qin

**Affiliations:** 1 School of Pharmaceutical Sciences, Zhejiang Chinese Medical University, Hangzhou, China; 2 The First Affiliated Hospital of Zhejiang Chinese Medical University, Hangzhou, China; 3 Institute of Botany, Jiangsu Province and Chinese Academy of Sciences, Nanjing, China; Foshan University, CHINA

## Abstract

Styrax, the balsam refined from the trunk of *Liquidambar orientalis* Mill. has a variety of applications in the perfumery and medical industry, especially for use in traditional Chinese medicine. However, the resources of styrax are in shortage due to being endangered of this plant. Grafting can improve the adaptability of plants to unfavorable environmental conditions. We tried to graft the *L*. *orientalis* Mill. on *L*. *formosana* Hance which was widely distributed in Jiangsu and Zhejiang provinces of China in an attempt to obtain styrax from grafted *L*. *orientalis* Mill. (grafted styrax, SG). Whether SG can become an alternative application of commercially available styrax (SC) need be further investigated. The components of SG were analyzed by GC-MS, and the results showed that the chromatograms of SG, SC, and styrax standard (SS) were consistent. The ration of 12 major chemical components based peak area in SG, SC, and SS were 93.95%, 94.24%, and 95.86% respectively. The assessment of toxicity, antithrombotic activity, and myocardial infarction protection of SG and SC was evaluated by using the zebrafish model, the results showed that SG and SC have the similar toxicological properties as evidenced by acute toxicity test, developmental toxicity and teratogenicity, and long-term toxicity test. Both SG and SC significantly decreased the thrombosis and increased blood flow velocity of zebrafish induced by adrenaline hydrochloride, inhibited myocardial apoptosis, myocardial infarction and myocardial inflammation in zebrafish induced by isoproterenol hydrochloride. Moreover, SG had an obvious improvement effect on cardiac output, while SC has no effect. Collectively, SG is similar to SC in chemical composition, toxicological properties, antithrombotic activity, and myocardial infarction protection effects, and may be used as a substitute for styrax to reduce the collection for wild *L*. *orientalis* Mill. and increase the available styrax resources.

## Introduction

Styrax, also named Suhexiang in China, is the balsam refined from the trunk of *Liquidambar orientalis* Mill. (Hamamelidaceae) [1], which is an endangered species and native to southwest Turkey (Türkiye) and Rhodos Island in Greece [2, 3]. Styrax is not only used in the perfumery industry, soap making, varnishes, and some medical applications, and also used externally as an antiseptic and parasiticide for skin disorders such as scabies and fungal illnesses, and used internally for upper respiratory illnesses such as asthma and bronchitis [1, 4, 5]. Styrax has been recorded in Pharmacopoeia of China and the United States Pharmacopoeia 43 [6, 7], and has been widely used in traditional Chinese medicine for its various pharmacological effects, including increasing coronary flow, improving microcirculation, lowering blood lipids and raising high-density lipoprotein, regulation of blood-brain barrier, anti-myocardial ischemia, anti-arrhythmia, anti-thrombosis, and anti-platelet aggregation [8–10], and often used for the treatment of angina pectoris in coronary heart disease and cerebral ischemia, abdominal pain, hypochondriac pain, and pinnacle headaches caused by qi stagnation, blood stasis, and cold clotting. Thrombosis, which is the leading cause of cardio-cerebrovascular diseases, and mainly induced by hypertension, high levels of low-density lipoprotein (LDL) and cholesterol [11]. Styrax demonstrates significantly anti-thrombosis activity, and is widely used in some Chinese patient medicines, for example, Guanxin Suhe Pill, Shexiang Baoxin Pill, Su Bing Dripping Pill, and Suhexiang Pill for the prevention and treatment of cardio-cerebrovascular diseases [12–14]. Therefore, styrax is the one of most important medicinal materials in Chinese patient medicines.

Considering the heavy demand for styrax in traditional Chinese medicine, and *L*. *orientalis* Mill. has been successfully introduced into botanical gardens in the United Kingdom, the United States and Belgium, some institutes in China tried to conduct introduction and cultivation of *L*. *orientalis* Mill, and found that it is not suitable for growth in China due to climate restrictions. Moreover, the Turkish customs banned the export of styrax in 2011, and the pharmaceutical enterprises of China should import styrax from Indonesia, Honduras, and England for medicinal purpose use [15]. Therefore, it is an urgent need to look for substituent resources of styrax.

Grafting is an ancient horticultural practice for asexual plant propagation that establishes a dual plant system by joining the scion of one plant with the rootstock of another plant [16, 17]. The rootstock can benefit the scion plant by enhancing its resistance to biotic and abiotic stresses and improving desirable agronomic traits, adapting scion cultivars to unfavorable environmental conditions [18–20]. Since the 1980s, in order to satisfy the medicinal requirement for styrax, we tried to graft the *L*. *orientalis* Mill. on *L*. *formosana* Hance widely distributed in Jiangsu and Zhejiang provinces of China to collect the resin in an attempt to substitute commercial styrax. The buds of *L*. *orientalis* Mill. as scions and *L*. *formosana* Hance as rootstocks were grafted and cultivated in Nanjing of Jiangsu province, Cangwu of Guangxi province, Changning of Yunnan province, Fuyang, Jinyun, Jiashan, and Tonglu of Zhejiang province in China. These grafted *L*. *orientalis* Mill. had a high survival rate and high-stress resistance, grew healthily, and increased the balsam yield significantly. Grafted *L*. *orientalis* Mill. can produce styrax after 5–10 years (named grafted styrax, SG), and our previous investigation demonstrates that grafted styrax had similar chemical profiles to commercial products. However, whether grafted styrax can substitute commercial styrax (SC) for use in Traditional Chinese Medicine (TCM) remains unclear, so their chemical constituents and toxicological and pharmacological properties should be further evaluated by comparison with commercial products.

The accumulating investigation exhibits that adult zebrafish is an ideal experimental animal with the advantages of solid fecundity, rapid development, short sexual maturity, embryo development *in vitro*, and easy observation and has been widely used to evaluate the toxicological and pharmacological properties of drugs [21]. In an attempt to clarify whether grafted styrax can substitute commercial styrax, we conducted the evaluation of grafted styrax by comparison with commercial styrax in chemical constituents, and toxicological and pharmacological properties relevant to its clinical application in the zebrafish model. We found that grafted styrax had a similar chemical profile and toxicological and pharmacological properties to commercial products, suggesting that grafted styrax can be used as a substitute for commercial styrax in TCM.

## Materials and methods

### Materials and reagents

The styrax standard (SS) was obtained from China National Institute for Food and Drug Control in Beijing, China (Batch number 120931–201804). The commercially available styrax (SC) was purchased from Bozhou medicinal material market (Anhui, China) and identified by Professor Lu-Ping Qin of Zhejiang Chinese Medical University. The grafted styrax (SG) was collected from grafted *L*. *orientalis* Mill. grow in the Institute of Botany, Jiangsu Province and Chinese Academy of Sciences (Nanjing, China), and prepared according to the description in the literature [22]. Cinnamyl cinnamate (≥99% HPLC; Lot No. H17AQ34087) and 3-phenylpropyl cinnamate (≥99% HPLC, Lot No. A16A11E111321) were purchased from Yuanye Bio-Technology Co., Ltd. (Shanghai, China). Compound Danshen Dropping Pills (CDDP) were supplied by Tasly Holding Group Co. Ltd. (Lot No. 190203, Tianjin, China). Isoproterenol hydrochloride was from Tixiai (Shanghai) Chemical Industry Development Co., LTD. (Lot No. Kiwnh-os, Shanghai, China). Acridine Orange (AO, Lot No. C12894919) and adrenalin hydrochloride (Lot No. C11075252) were obtained from Maclin (Shanghai, China). O-dianisidine was from Sigma-Aldrich (Lot No. MKCC7501, MO, USA).

### Chemical constituents analysis

50 mg styrax was dissolved in 5 mL of anhydrous ethanol in the centrifuge tube. 3 mg cinnamyl cinnamate and 3-phenylpropyl cinnamate as reference substances were weighed precisely and dissolved with 1 mL anhydrous ethanol, respectively. All sample solutions were filtered by a membrane (0.22 μm) and used as the test solution. The chemical constituents were analyzed by using a gas chromatography-mass spectrometry (GC-MS) System. GC-MS System consisted of a 7890B gas chromatograph (Agilent Technologies, CA, USA), an Agilent 5977A MSD (Agilent Technologies), and a 7693 auto-sampler (HP-5, 30 m × 0.25 mm, 0.25μm film thickness, Agilent Technologies). The mass spectral scan range was m/z 35–500. The GC was operated in splitting injection mode with a nitrogen flow rate of 0.7 mL/min and 1 μL injection volume, the split ratio was 10:1. The MS was operated in the electron ionization (EI) mode with an ionization voltage of 70 eV, a source temperature of 230 °C, and the transfer line at 280 °C. The temperature program consisted of an initial hold at 60 °C for 5 min, ramped up to 280 °C at a rate of 4 °C/min followed by a hold at 280 °C for 10 min. The chemical compounds were identified against the characteristic peaks in the GC-MS total ion flow chromatogram by searching the NIST14 database of the Public Platform of Pharmaceutical Research Center, Academy of Chinese Medical Science, Zhejiang Chinese Medical University, and comparing them with the controls. In addition, the content of cinnamic acid after alkali hydrolysis of styrax was determined by high performance liquid chromatography according to the Chinese Pharmacopoeia (general rule 0512).

### Zebrafish experiments

The zebrafish were maintained in fish water at 28 °C (0.2% Instant Ocean Salt in deionized water, pH 6.9–7.2, conductivity 450–550 S/cm, pH 6.5–8.5 and hardness 50–100 mg/L CaCO3), bred and supplied by the Fish Farming Center of Hunter Biotechnology, Inc., and the license number for laboratory animals is SYXK (Zhejiang) 2012–0171, SYXK (Zhejiang) 2022–0003, SYXK (Zhejiang) 2022–0004. This study was approved by the Institutional Animal Care and Use Committee (IACUC) at Hunter Biotechnology, Inc. and the IACUC approval number was 001458. The zebrafish facility and the laboratory at Hunter Biotechnology, Inc., are accredited by the Association for Assessment and Accreditation of Laboratory Animal Care (AAALAC) International. During the experiment, the general status, behavior and abnormal phenotype of zebrafish were observed daily. The zebrafish were administrated with 0.08 mg/mL tricaine to anesthetize, and then their body weight was weighed every 15 days. At the end of the experiment, the zebrafish were sacrificed by soaking into a mixture of ice and water for more than 15 minutes till the they became completely unconscious, and then placed in 300mg/L tricaine solution for 15–20 minutes to minimize their possible suffering.

### Acute toxicity test

The zebrafish have completed the development of various organs at 48–120 hour post fertilization (hpf), and are qualified to be used for the evaluation of acute toxicity of drugs [23, 24]. Hence, 48 hpf wild-type AB zebrafish were used and randomly transferred into the six-well plates with 30 fish per well, containing 3 mL of fresh fish water. The fish were exposed to series concentrations of SG or SC (3.91, 7.81, 15.6, 31.2, and 62.5 μg/mL) continuously for 72 h. The water was refreshed every day. During the exposure period, the lethality was recorded according to the Organisation for Economic Co-operation and Development (OECD) guidelines [25]. The number of dead larvae at each concentration was plotted and a concentration-response curve was fitted. The maximum non-lethal concentration (MNLC) and 10% lethal concentration (LC_10_) were calculated from independent experiments performed in triplicate. According to the 72 h MNLC and LC_10_ value, four concentrations of SG and SC (1.95, 3.91, 7.81, and 15.6 μg/mL) were set for acute toxicity evaluation. The fish were anesthetized and images of the eye area, lower jaw area, liver area, intestine area, heart size, yolk sac area, and swim bladder size were captured using optical microscopy (SZX7, Olympus, Tokyo, Japan), and analyzed using NIS-Elements D 3.20 software (Nikon, Tokyo, Japan). The relative values were calculated by the following formula: relative values = individual values/mean value of the control group.

### Developmental toxicity and teratogenicity test

The zebrafish often complete various organs development within 24–72 hours, such as embryonic development within the first 72 hours, cardiovascular system, intestine, liver and kidney development within 24–48 hours. So, the 6 hpf zebrafish are basically still in developmental stage of various organs, and often are applied to assess developmental toxicity and teratogenicity [23, 24, 26]. 6 hpf wild-type AB zebrafish were exposed to series concentrations of SG or SC (1.95, 3.91, 7.81, 15.6, 31.2, and 62.5 μg/mL) in 3 mL fresh fish water for 120 h. The water was refreshed every day. The MNLC and LC_10_ were calculated from independent experiments performed in triplicate. According to the 120 h MNLC and LC_10_ value, four concentrations of SG and SC (1.95, 3.91, 7.81, and 15.6 μg/mL) were set for developmental toxicity and teratogenicity evaluation. The organ morphological changes were observed as described in acute toxicity test.

### Long term toxicity test

The zebrafish was treated with SG or SC at 0.975, 1.95, 3.90, 7.80 μg/mL for 14 days, the minimal toxic concentrations (MTCs) of both SG and SC were 1.95 μg/mL. In addition, SG and SC at 1.95 μg/mL had no toxicological effects on zebrafish, and should have significant effects on antithrombotic and myocardial protection. Hence, SG and SC at 1.95μg/mL were performed to evaluate their long-term toxicity. 5 mpf wild-type AB zebrafish were performed and randomly transferred into the beaker with 10 fish per beaker, sex in half, containing 4 L fresh fish water. The fish were exposed to 1.95 μg/mL SG or SC for 3 months, and the body weight was measured every 15 days. After 3 months, the zebrafish were anesthetized, fixed, dehydrated, embedded, and sliced, and then HE staining was performed. Histopathological alterations were observed under optical microscopy (SZX7, Olympus, Tokyo, Japan).

### Antithrombotic assay

The adrenaline hydrochloride causes decreased blood flow velocity and increased platelet aggregation in zebrafish, and then induce the thrombosis. Hence, adrenaline hydrochloride is often used to establish zebrafish model of thrombosis. In addition, Compound Danshen Dripping Pill (CDDP) has been widely used to treat cardiovascular diseases, including coronary arteriosclerosis, myocardial ischemia-reperfusion injury, myocardial infarction and angina pectoris, and exhibits definitely therapeutic effects. In the preliminary experiments, we observed the protective effects of CDDP at concentrations of 2000, 1000, 500, 250, 125, 62.5, and 31.2 μg/mL on myocardial apoptosis in zebrafish, and found that 62.5 μg/mL of CDDP had most significant effects. Therefore, 62.5 μg/mL CDDP was used as the positive control in the evaluation of antithrombotic activity, and myocardial infarction protection of styrax. 72 hpf translucent Albino strain zebrafish with melanin allele mutation were used and randomly transferred into the six-well plates with 30 fish per well containing 3 mL fresh fish water. The fish were co-exposed to series concentrations of SG or SC (0.977, 1.95, and 3.91μg/mL) and 62.5 μg/mL CDDP. The normal group and model group were set up at the same time. In addition to the normal group, the other groups were given 25 mM adrenaline hydrochloride in a water-soluble manner to establish a zebrafish thrombosis model for 2 days. The blood flow velocity of the tail was analyzed by recording zebrafish blood flow video using the heartbeat blood flow analysis system (ZebraBlood 3.3, ViewPoint Life Sciences, Lyon, France). O-dianisidine can be coupled with ferrous ions to specifically label hemoglobin, thus indicating the distribution of red blood cells [27–29]. After staining with O-dianisidine for 15 minutes in dark, the incidence of tail stasis was initially determined by screening, and 10 fish were randomly selected from each group of experiments to take photos under optical microscopy. The data collected from the photos were analyzed with NIS-Elements D 3.20 software, and the blood stasis in the zebrafish tail was analyzed and counted.

### Evaluation of myocardial apoptosis

48 hpf wild-type AB zebrafish were exposed to series concentrations of SG or SC (0.977, 1.95, and 3.91μg/mL) and 62.5 μg/mL CDDP continuously for 1 h. The normal group and model group were set up at the same time. Then, the drug groups and the model group were treated with 50 mg/mL isoproterenol hydrochloride for 5 h. Myocardial apoptotic cells were identified by OA staining for 30 minutes in dark, and 10 fish were randomly selected from each group of experiments to take photos under the fluorescence microscope (AZ100, Nikon, Tokyo, Japan). The data collected from the photos were analyzed with NIS-Elements D 3.20 software, and the fluorescence intensity of apoptotic cells in the heart of zebrafish was analyzed and counted.

### Measurement of myocardial function

48 hpf wild-type AB zebrafish were exposed to series concentrations of SG or SC (0.977, 1.95, and 3.91μg/mL) and 62.5 μg/mL CDDP continuously for 4 h and then were treated with 50 mg/mL isoproterenol hydrochloride for 1 h. The 10 fish in each experimental group were randomly selected to record and analyze the blood flow velocity and cardiac output by using the heartbeat blood flow analysis system (ZebraBlood 3.3, ViewPoint Life Sciences, Lyon, France) to record blood flow to the torso near the heart.

### Determination of myocardial infarction

Zebrafish embryos were treated with analogs previously described [30]. Briefly, Tg (*cmlc*: EGFP) embryos were collected and embryos were allowed to develop for 48 h. These transgenic heart green fluorescent zebrafish were used for further experiments. The fish treatment was the same as the detection of myocardial function. The zebrafish were anesthetized and images were captured using the fluorescence microscope. The myocardial infarction was analyzed using NIS-Elements D 3.20 software. 10 fish were randomly selected in each experimental group.

### Quantitative real-time-polymerase chain reaction (qRT-PCR) analyses

Transgenic Tg (MPO: EGFP) zebrafish showed green fluorescence specifically expressed in neutrophils which evaluates the neutrophil numbers. The method was as previously described [31, 32]. 48 hpf transgenic neutrophil green fluorescent zebrafish were co-exposed to drugs and 50 mg/mL isoproterenol hydrochloride for 1 day. The drugs were including series concentrations of SG or SC (0.977, 1.95, and 3.91μg/mL) and 62.5 μg/mL CDDP. The zebrafish were anesthetized and images were captured using the fluorescence microscope. The neutrophil numbers were analyzed using NIS-Elements D 3.20 software. 10 fish were randomly selected in each experimental group.

Total RNA was isolated from transgenic neutrophil green fluorescent zebrafish using RNA-easy Isolation Reagent (Vazyme Biotech, Nanjing, China). Briefly, 30 fish were homogenized with 300 μL of lysis buffer using a homogenizer, centrifuged at 12,000 × *g* for 15 min, and the supernatant was transferred to a new tube. The same volume of isopropanol was added to precipitate the RNA. After being centrifuged at 12,000 × *g* for 10 min, the pellet was washed with 75% ethanol. Three parallel experiments were set up. The RNA was dissolved with RNase-free ddH_2_O and the purity was checked using RNA-quick Purification Kit (Yishan Biological, Shanghai, China). A total of 2 μg RNA was reverse transcribed into cDNA by using a Fast Quant RT kit (Tiangen Biotech, Beijing, China). Gene expressions were detected by qRT-PCR using iTaq Universal SYBR Green (Bio-Rad, Hercules, CA, USA) on the CFX Connect Real-Time PCR Detection System (Bio-Rad, CA, USA). The primer sequences were referred to other studies and listed in Table 1 [33–41]. The relative expression levels of the genes were normalized to β-actin and calculated using the 2^-ΔΔCT^ method.

**Table 1:** Sequence of primers used in this study.

Gene	Forward primer	Reverse primer
**β-actin**	5’-GCTCTCTTCCAGCCTTCCTT-3’	5’-GAAGGTGGTCTCGTGGATACC-3’
**IL-1β**	5’-GTCACACTGAGAGCCGGAAG-3’	5’-GCAGGCCAGGTACAGGTTAC-3’
**TNF-α**	5’-AGGAGAGTTGCCTTTACCGC-3’	5’-AATGGATGGCAGCCTTGGAA-3’
**tnnt2α**	5’-GTCTGCACTTCGGCGGTTACA-3’	5’-CTGAGAGCAGATTCATTGGC-3’
**COX-2**	5’-TGTTTTGAACGAGCGGAGTT-3’	5’-CAAAGTTGCACATCGATCACA-3’
**ANP**	5’-CTTCCACATCCTGGGACAGAGA-3’	5’-TGCAGCTAACCTTTTTTAGAGTTGC-3’
**BNP**	5’-AAGAGCAGCCCGATACTTACCT-3’	5’-TCCCAAAGACGACATTGAACC-3’

### Statistical analysis

All data in this study were presented as mean ± standard error (SE), and analyzed by one-way analysis of variance (ANOVA) followed by Dunnett’s multiple comparisons tests using SPSS26.0. *P* < 0.05 was considered significant.

## Results and discussion

### Chemical profiles of SG and SC

**Fig 1:**
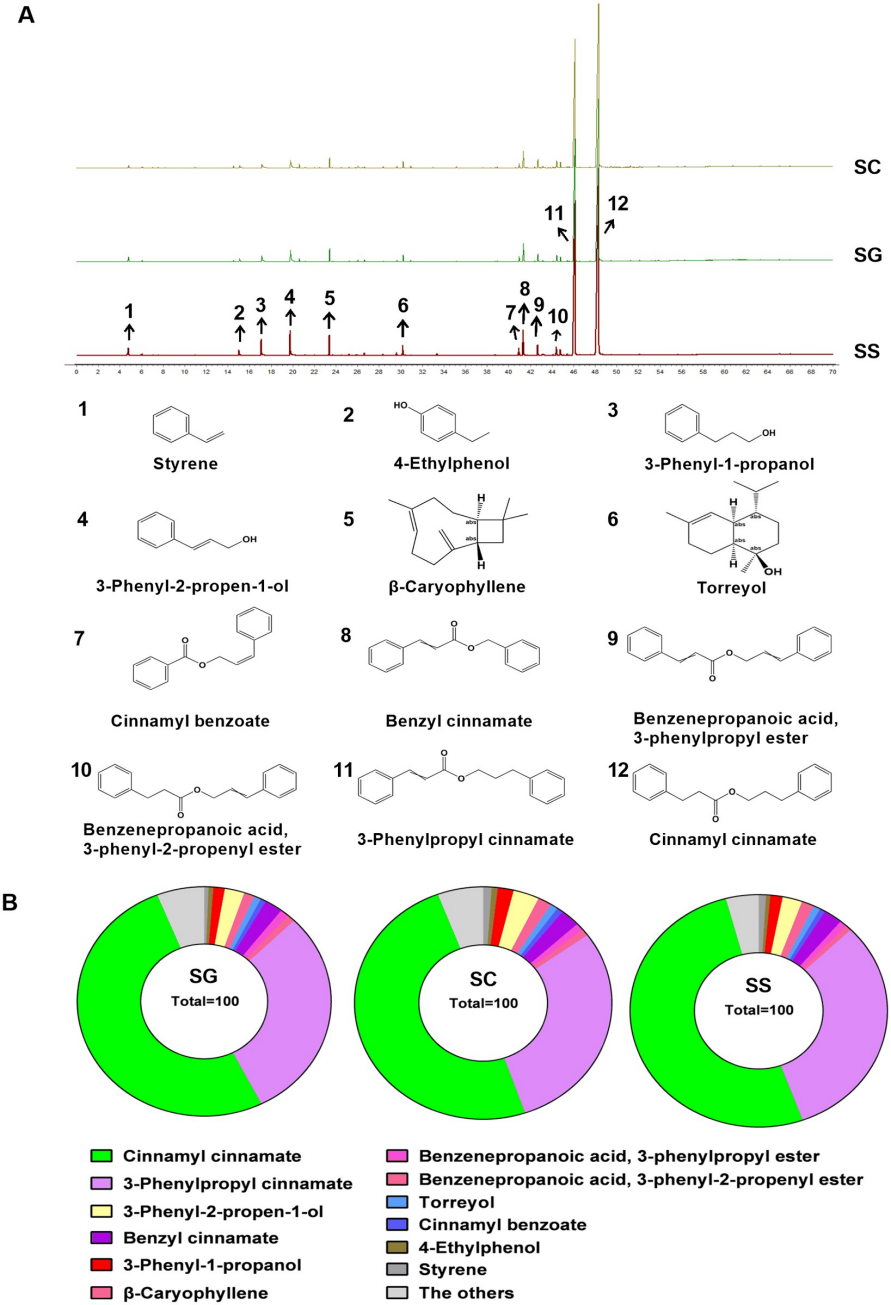
Identification of the chemical composition of SG, SC, and SS by GC-MS. (A) GC-MS extracted ion chromatograms for SG, SC, and SS under full scan mode. (B) The proportion of chemical components in SG, SC, and SS, respectively.

GC-MS analysis was used to identify the chemical constituents of SG and SC. As shown in Fig 1A, the characteristic chromatograms of SG and SC were basically consistent with that of the SS. The three chromatograms were imported into the similarity evaluation system for the chromatographic fingerprint of TCM to generate the control chromatogram. The similarity between the chromatogram samples and the control chromatogram was evaluated by the similarity calculation function. The similarity of SG and SC relative to the control chromatogram was nearly 1.0, indicating that SG had similar chemical composition to SC. The compounds represented by characteristic peaks in GC-MS total ion current chromatogram were identified by searching the NIST database and comparing them with the control substance. A total of 59 chemical components in styrax were identified (S1 Table), mainly including aromatic compounds and terpenoids [11]. 24 common chemical components were found in three samples (Table 2). The proportion of the 12 main compounds, including styrene (peak 1), 4-ethylphenol (peak 2), 3-phenyl-1-propanol (peak 3), 3-phenyl-2-propen-1-ol (peak 4), β-caryophyllene (peak 5), torreyol (peak 6), cinnamyl benzoate (peak 7), benzyl cinnamate (peak 8), benzenepropanoic acid, 3-phenylpropyl ester (peak 9), benzenepropanoic acid, 3-phenyl-2-propenyl ester (peak 10), 3-phenylpropyl cinnamate (peak 11) and cinnamyl cinnamate (peak 12) based on peak area was 93.95% in SG, 94.24% in SC, and 95.86% in SS, respectively (Fig 1B). Cinnamyl cinnamate and 3-phenylpropyl cinnamate were the highest contents in these three samples (Fig 1A) and were representative chemical components of styrax, which is consistent with the results of previous studies [22]. According to reports, the chemical constituents isolated from styrax are mainly terpenoids, aromatic organic acids, and their derivatives [11]. Sun *et*.*al* identified 18 compounds in commercial styrax, including styrene, pinene, benzyl alcohol, acetophenone, 4-ethylphenol, benzoic acid, 3-phenyl-1-propanol, cinnamaldehyde, cinnamyl alcohol, β-caryophyllene, cinnamic acid, benzyl benzoate, benzyl cinnamate, phenylpropyl phenylpropionate, cinnamyl phenylpropionate, 3-phenylpropyl cinnamate, cinnamyl cinnamate, and oleanonic acid [22]. The results presented in this paper show that except for benzoic acid and oleanonic acid, the other 16 components were also found in SG, further exhibiting that SG had similar chemical constituents to SC.

**Table 2:** The common chemical components of SG, SC, and SS.

Compound	CAS	Molecular formula	SG			SC			SS		
			RT	Peak area (%)	NIST match (similarity, %)	RT	Peak area (%)	NIST match (similarity, %)	RT	Peak area (%)	NIST match (similarity, %)
**Styrene**	100-42-5	C_8_H_8_	4.78	0.54	98.99	4.79	1.05	98.47	4.80	0.90	99.03
**Cyclofenchene**	488-97-1	C_10_H_16_	6.05	0.27	97.35	6.06	0.30	97.42	6.07	0.28	96.93
**Benzyl alcohol**	100-51-6	C_7_H_8_O	9.77	0.06	91.08	9.78	0.11	94.77	9.86	0.03	86.36
**DL-1-Phenethylalcohol**	98-85-1	C_8_H_10_O	10.78	0.03	95.15	10.79	0.06	95.59	10.79	0.03	89.42
**Acetophenone**	98-86-2	C_8_H_8_O	10.88	0.09	96.20	10.89	0.12	94.19	10.92	0.06	91.69
**4-Ethylphenol**	123-07-9	C_8_H_10_O	15.04	0.62	96.35	15.08	0.77	96.73	15.03	0.55	96.96
**3-Phenyl-1-propanol**	122-97-4	C_9_H_12_O	17.14	1.45	97.89	17.18	2.00	97.62	17.09	1.54	98.57
**Cinnamyl alcohol**	4407-36-7	C_9_H_10_O	19.86	2.52	97.56	19.91	3.28	97.43	19.78	2.48	97.99
**α-Cubenene**	17699-14-8	C_15_H_24_	21.14	0.09	96.11	21.15	0.08	96.33	21.14	0.06	94.00
**β-Caryophyllene**	87-44-5	C_15_H_24_	23.42	1.36	99.01	23.44	1.76	98.88	23.40	1.61	99.16
**β-Copaene**	18252-44-3	C_15_H_24_	23.71	0.06	96.41	23.72	0.07	96.52	23.70	0.07	93.00
**α-Humulene**	6753-98-6	C_15_H_24_	24.47	0.05	92.86	24.48	0.06	95.35	24.46	0.05	91.50
**(+)-δ-cadinene**	483-76-1	C_15_H_24_	26.64	0.18	93.68	26.65	0.26	95.34	26.63	0.22	95.17
**Cubenene**	29837-12-5	C_15_H_24_	26.90	0.03	91.32	26.90	0.05	95.55	26.89	0.03	90.26
**Caryophyllene oxide**	1139-30-6	C_15_H_24_O	28.37	0.24	98.00	28.38	0.15	97.11	28.36	0.11	92.55
**Torreyol**	19435-97-3	C_15_H_26_O	30.24	0.89	96.51	30.25	0.95	95.91	30.20	0.86	95.96
**Benzyl benzoate**	120-51-4	C_14_H_12_O_2_	33.37	0.03	86.67	33.39	0.03	88.33	33.37	0.18	97.20
**Cinnamyl benzoate**	117204-78-1	C_16_H_14_O_2_	40.97	0.70	92.90	40.99	0.80	93.26	40.94	0.70	96.36
**Benzyl cinnamate**	103-41-3	C_16_H_14_O_2_	41.41	2.31	97.58	41.43	2.46	97.62	41.34	2.35	98.24
**Benzenepropanoic acid,3-phenylpropyl ester**	60045-27-4	C_18_H_20_O_2_	42.72	1.12	94.86	42.72	1.05	94.86	42.68	0.96	96.21
**Bornyl Cinnamate**	6330-67-2	C_19_H_24_O_2_	44.10	0.12	88.68	44.10	0.10	87.81	44.09	0.08	91.81
**Benzenepropanoic acid, 3-phenyl-2-propenyl ester**	28048-98-8	C_18_H_18_O_2_	44.45	1.01	94.14	44.46	0.92	94.81	44.42	0.72	97.00
**3-Phenylpropyl cinnamate**	122-68-9	C_18_H_18_O_2_	46.36	30.04	98.68	46.38	29.68	98.68	46.15	31.79	98.73
**Cinnamyl cinnamate**	122-69-0	C_18_H_16_O_2_	48.66	51.39	96.27	48.67	49.52	96.27	48.34	51.40	96.38
**The others**				4.80			4.37			2.94	

Both United States Pharmacopoeia and China Pharmacopoeia stipulated the quality control standard of styrax [6, 7], involving origin, character, identification, inspection, and content determination, the content of total cinnamic acid after alkali hydrolysis is used as an indicator of quality control. SG and SC were analyzed according to the quality control standard of styrax in Chinese Pharmacopoeia 2020. The results showed that the content of cinnamic acid in SG and SC were respectively 22.05% and 21.89%, conforming to the quality control standard of Chinese Pharmacopoeia [6], nearly reaching the requirement of more than 25% in United States Pharmacopoeia. Sun determined the content of 3-phenylpropyl cinnamate and found the content of 3-phenylpropyl cinnamate reached to 14.4% ~ 17.9% in 18 batches of commercial styrax. Hence, Sun pointed out that the contents of cinnamyl cinnamate and 3-phenylpropyl cinnamate accounted for about 70% of chemical constituents in commercial styrax. The contents of cinnamyl cinnamate and 3-phenylpropyl cinnamate were about 81.43% in SG based on the area normalization method. Therefore, it can be concluded that the main chemical constituents of SG and SC are highly similar as evidenced by the chemical profile and the content of cinnamic acid, and grafting did not affect the chemical composition of styrax, and SG may be a good choice to substitute SC.

### Toxicological properties of SG and SC

**Fig 2:**
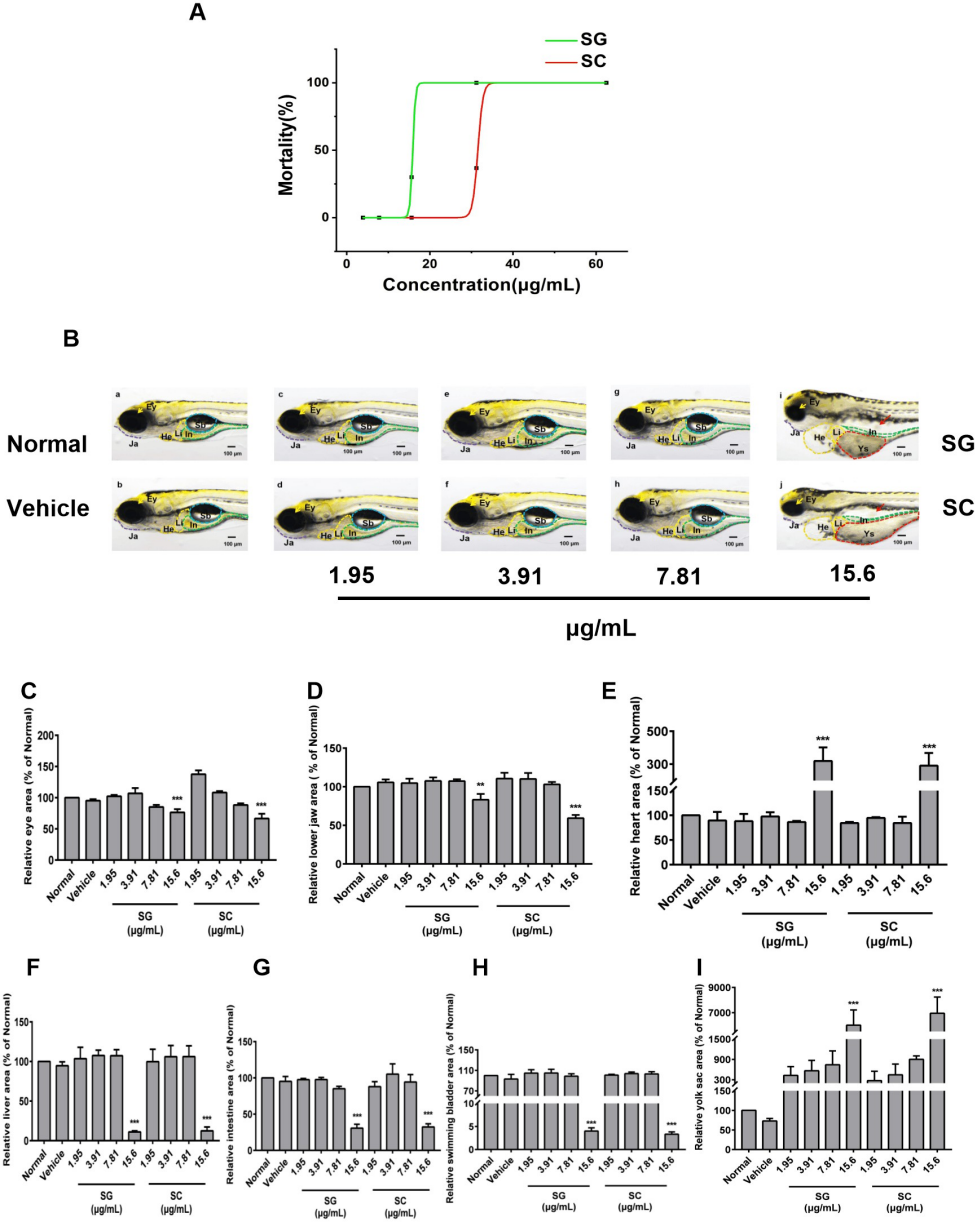
Acute toxicity assessment. (A) The dose-death curve of SG and SC. The 48 hpf zebrafish were exposed to 3.91, 7.81, 15.6, 31.2, and 62.5 μg/mL SG or SC for 72 h. (B) Representative picture of zebrafish from the normal group (untreated) and vehicle groups (0.5% ethanol absolute) and series concentration of SG and SC. (C-I) The relative area of the normal group of each organ was measured by NIS-ElementsD 3.20 software. (C) The eye (Ey), (D) The lower jaw (Ja), (E) The heart (He), (F) The liver (Li), (G) The intestine (In), (H) The swimming bladder (Sb), (I) The yolk sac. Images were captured at 400× magnification. Scale bars: 100 μm. The results are represented as a mean ± SE (n = 3). ** *P* < 0.01, *** *P* < 0.001 vs. vehicle groups.

The zebrafish are highly similar to mammals in physiological characteristics, and has been widely applied to evaluate the toxicological properties of drugs [42]. In the present study, the zebrafish were used to evaluate the safety of SG by comparison with SC. The 48 hpf zebrafish were exposed to different concentrations of SG and SC (1.95, 3.91, 7.81, and 15.6 μg/mL) for 72 h to compare their acute toxicity. As shown in Fig 2A, both SG and SC increased the mortality of zebrafish in a concentration-dependent manner. According to the Hill equation fitting, the maximum non-lethal concentration (MNLC) and 10% lethal concentration (LC_10_) of SG are 14.3 and 15.1 μg/mL respectively, while the MNLC and LC_10_ of SC are 26.4 and 29.2 μg/mL, respectively. SG less than 15.6 μg/mL did not cause the alteration in morphology and organs of zebrafish. Compared with the vehicle group, treatment zebrafish with SG and SC of 15.6 μg/ml caused a significant decrease in the area of eyes (Fig 2B and 2C), lower jaw (Fig 2B and 2D), liver (Fig 2B and 2E), intestine (Fig 2B and 2F) and swimming bladder (Fig 2B and 2G), and increase in the area of the heart (Fig 2B and 2H) and yolk sac edema (Fig 2B and 2I).

**Fig 3:**
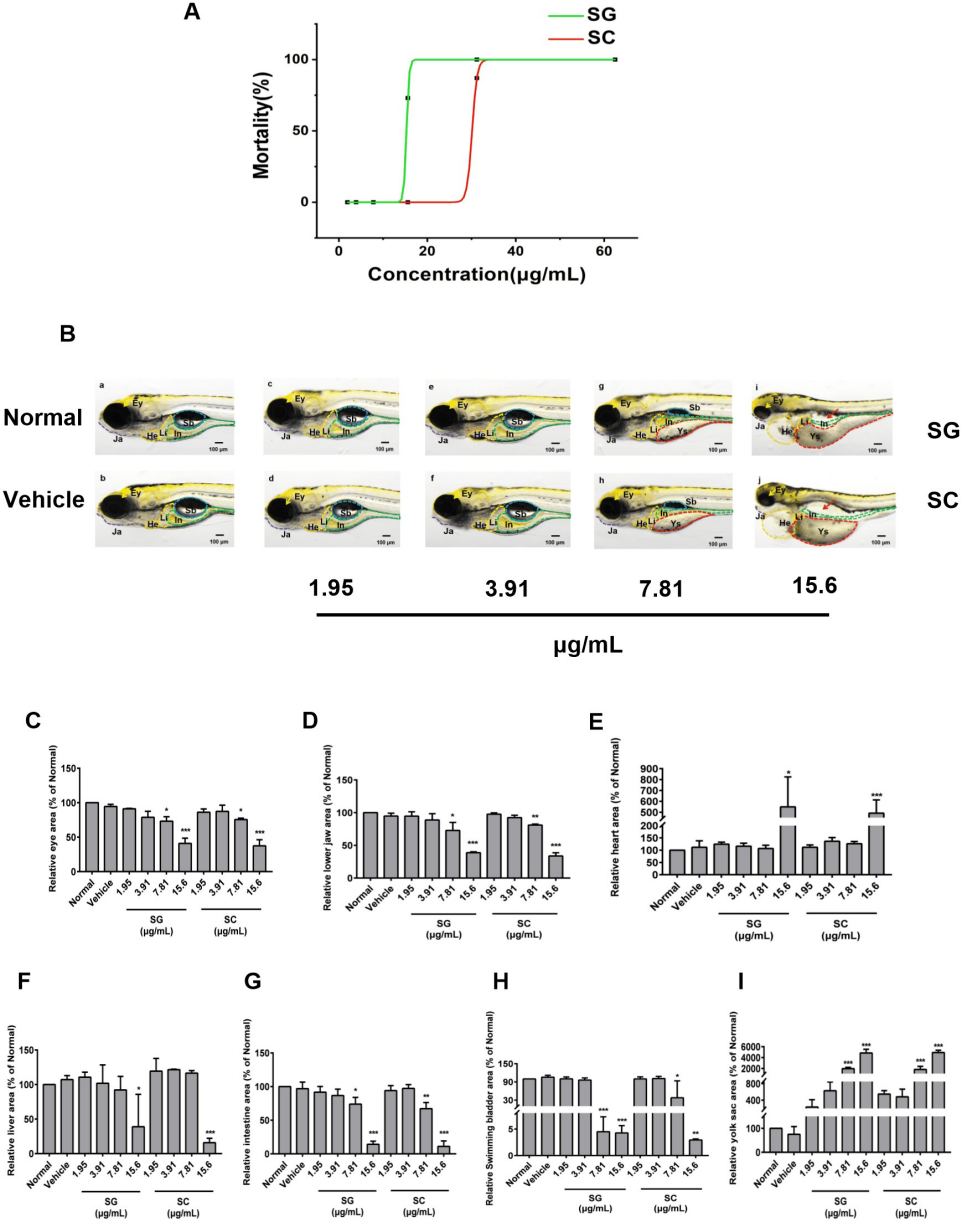
Evaluation of developmental toxicity and teratogenicity. (A) The dose-death curve of SG and SC. 6 hpf zebrafish were exposed to 1.95, 3.91, 7.81, 15.6, 31.2, and 62.5 μg/mL SG or SC continuously for 120 h. (B) Representative picture of zebrafish from the normal group (untreated) and vehicle groups (0.5% ethanol absolute) and series concentration of SG and SC. (C-I) The relative area of the normal group of each organ was measured by NIS-ElementsD 3.20 software. (C) The eye (Ey), (D) The lower jaw (Ja), (E) The heart (He), (F) The liver (Li), (G) The intestine (In), (H) The swimming bladder (Sb), (I) The yolk sac. Images were captured at 400× magnification. Scale bars: 100 μm. The results are represented as a mean ± SE (n = 3). * *P* < 0.05, ** *P* < 0.01, *** *P* < 0.001 vs. vehicle groups.

Then 6 hpf zebrafish were treated with different concentrations of SG and SC for 120 h to observe their developmental toxicity and teratogenicity. As shown in Fig 3A, both SG and SC increased the mortality of zebrafish in a concentration-dependent manner. The MNLC and LC_10_ of SG are 13.8 and 14.6 μg/mL respectively, while the MNLC and LC_10_ of SC are 27.2 and 28.6 μg/mL, respectively. SG and SC at concentration of 7.81 μg/mL showed developmental toxicity and teratogenicity, mainly manifested as the swim bladder became significantly smaller (Fig 3B and 3G) and the yolk sac became significantly bigger (Fig 3B and 3I). SG and SC at concentration of 15.6 μg/mL caused serious malformations in zebrafish, including smaller eyes (Fig 3B and 3C), shorter lower jaw (Fig 3B and 3D), hepatocytes structure disorder (Fig 3B and 3E), intestine (Fig 3B and 3F), smaller swim bladder (Fig 3B and 3G), pericardial effusion (Fig 3B and 3H) and yolk sac edema (Fig 3B and 3I).

**Fig 4:**
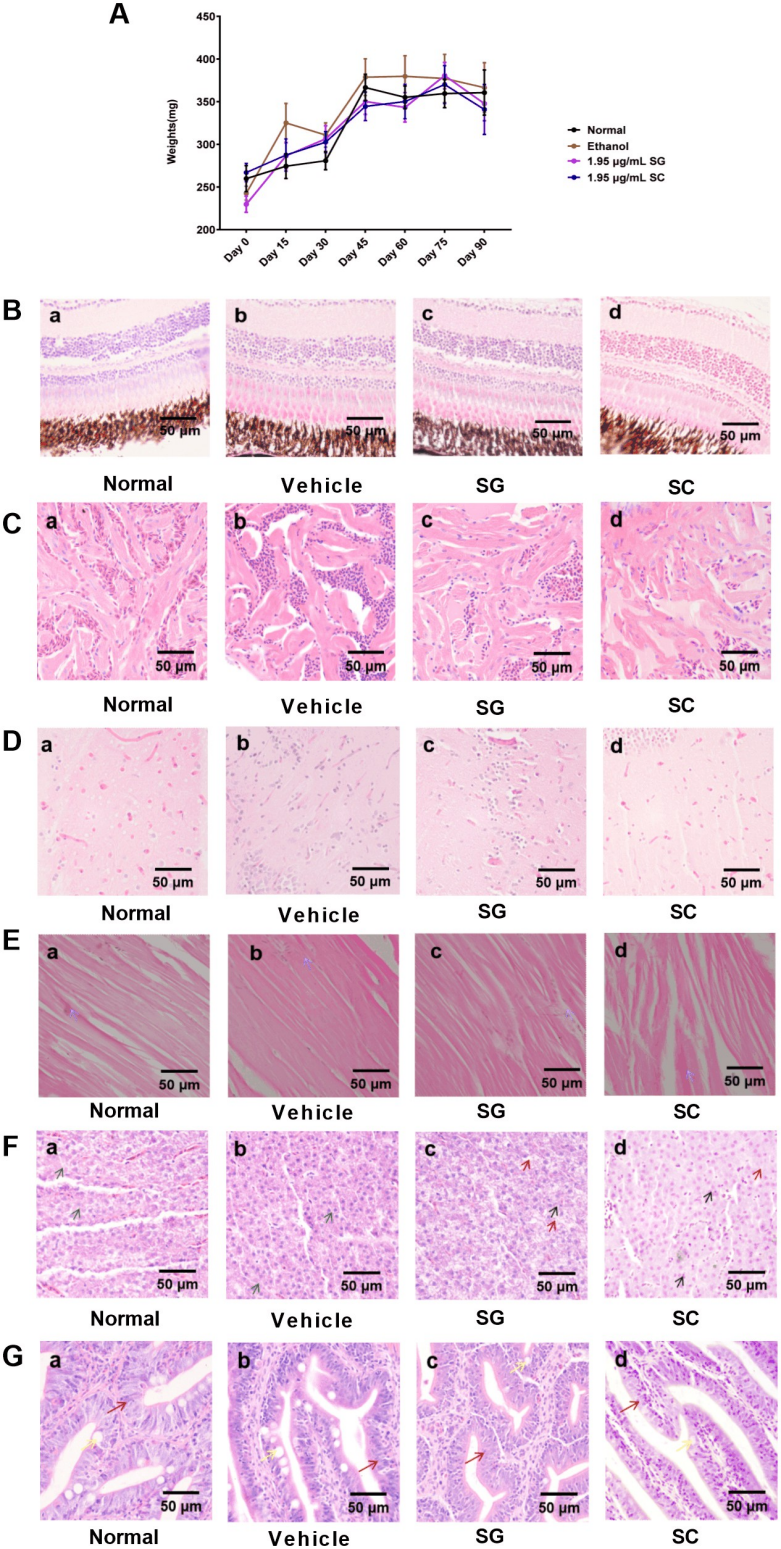
Long-term toxicity assessment. 5 mpf zebrafish were exposed to 1.95 μg/mL SG or SC continuously for 3 months. (A) The body weight. (B-G) Histopathological picture of the organs analyzed. (B) The eye, (C) The heart, (D) The brain, (E) The muscle, (F) The liver, (G) The intestine. H&E (400×). Scale bars: 50 μm.

5 mpf zebrafish were used to further evaluate the long-term toxicity of SG and SC. The results showed that there was no significant difference in the weight among SG, SC treatment group and vehicle group (Fig 4A), suggesting that SG and SC at concentration of 1.95 μg/mL did not inhibit the growth of zebrafish. The histopathological results showed that compared with the vehicle group, the hepatocytes were not closely arranged (Fig 4F), the intestinal villus was slightly shorter (Fig 4G) and the number of goblet cells was reduced (Fig 4G and 4I) in SG group and SC group. However, SG and SC had no effect on the eyes (Fig 4B), heart (Fig 4C), brain (Fig 4D), and muscle (Fig 4E) of zebrafish. These results suggest that the toxic target organs of SG and SC may be the liver and intestine, and had similar toxicological properties. These toxicological results also reminder us to monitor the liver and intestine injury in the clinical use of SG and SC.

### Antithrombotic activity of SG and SC

**Fig 5:**
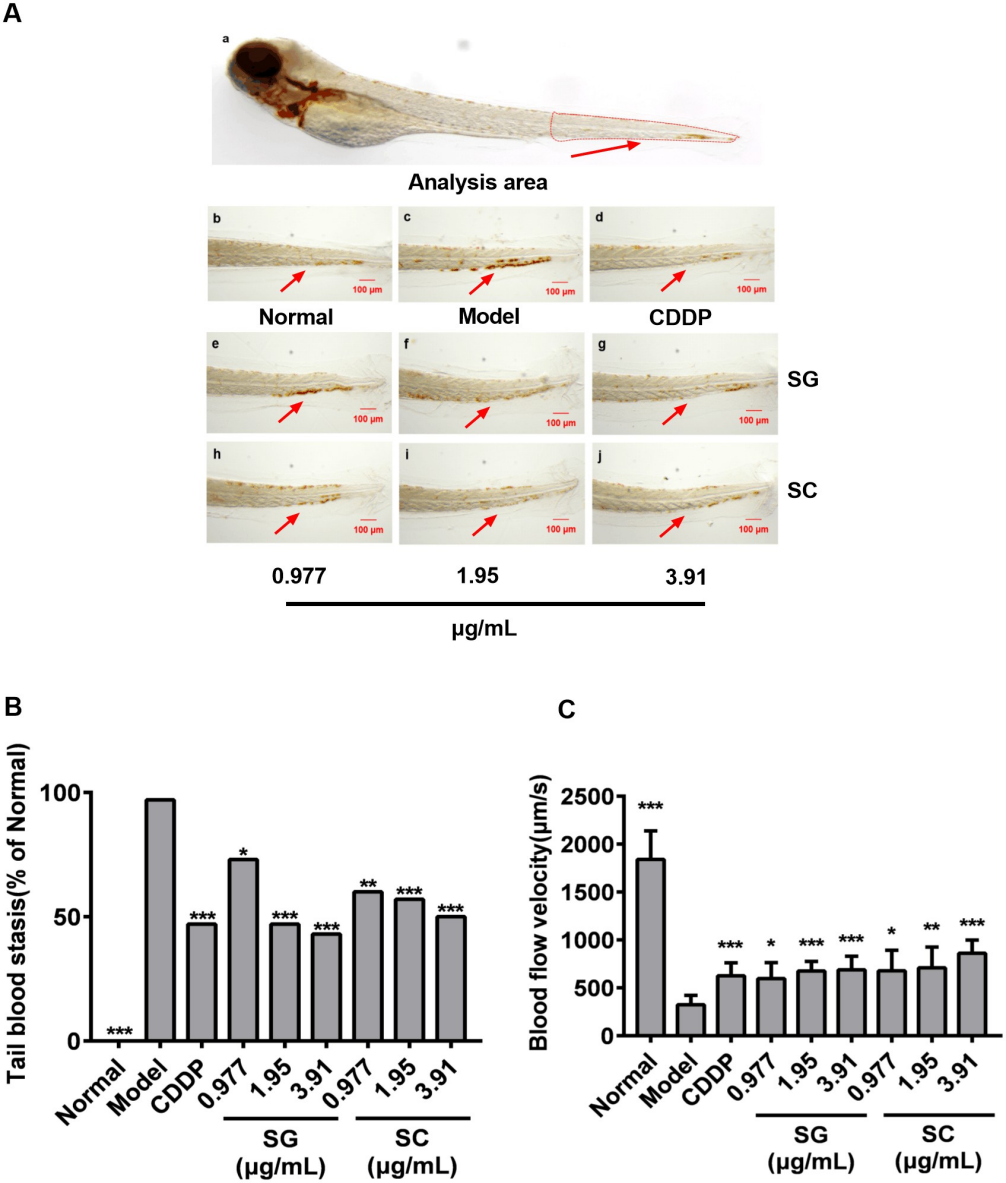
Antithrombotic activity of SG and SC. (A) Representative picture of zebrafish tail vein thrombosis. (B) The occurrence rate of tail blood stasis. (C) Blood flow velocity. Images were captured at 400× magnification. Scale bars: 100 μm. The results are represented as a mean ± SE (n = 10). * *P* < 0.05, ** *P* < 0.01, *** *P* < 0.001 vs. model groups.

Styrax is the crucial ingredient of TCM medicine that has been used to treat blood stasis syndrome, especially cardio-cerebrovascular diseases [13, 43, 44], and thrombus formation in blood vessels is the predominant cause of blood stasis syndrome. The thrombosis model of zebrafish was established by the administration of adrenaline hydrochloride to evaluate the antithrombotic activity of SG and SC. As shown in Fig 5A and 5B. Administration of adrenaline hydrochloride caused a significant increase in the incidence of congestion in the tail of zebrafish (Fig 5A and 5B) and a significant decrease in the blood flow rate (Fig 5C), while CDDP, SG, and SC significantly attenuated the incidence of tail congestion and enhanced the blood flow rate (Fig 5A–5C). There was no significant difference in reducing blood stasis and increasing the blood flow rate between SG and SC. Therefore, SG was the same effectively as SC in prevention the incidence of congestion in the tail of zebrafish, and can be used in the TCM formula for the treatment of blood stasis syndrome.

### Effects of SG and SC on myocardial infarction induced by isoproterenol in zebrafish

**Fig 6:**
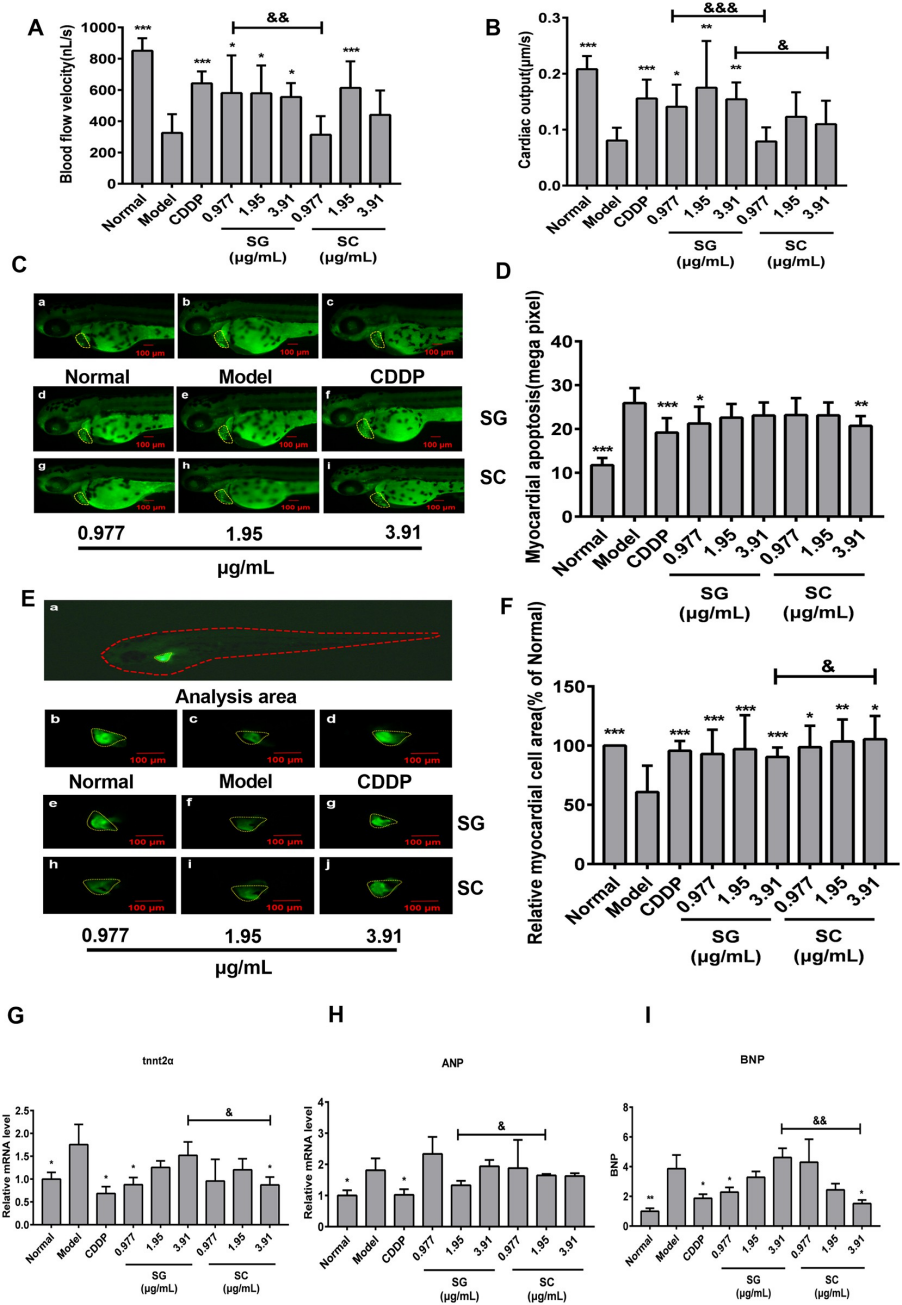
The myocardial protective effects of SG and SC. (A) Blood flow velocity. (B) Cardiac output. (C-D) Fluorescence micrographs of apoptotic cells in zebrafish. (E-F) Phenotypes of fish of Tg (cmlc: EGFP) lines. (G-I) qRT-PCR analysis was performed to measure the heart function-related genes tnnt2α, ANP, and BNP in Tg (MPO: EGFP) zebrafish. Images were captured at 400× magnification. Scale bars: 100 μm. The results are represented as a mean ± SE (n = 10). * *P* < 0.05, ** *P* < 0.01, *** *P* < 0.001 vs. model groups. ^&^ *P* < 0.05, vs.SC.

Styrax has been used for the treatment of cardiovascular diseases in China and other Asia countries, such as myocardial infarction [45, 46]. The myocardial infarction model of zebrafish was established by isoproterenol hydrochloride to compare the protective effects of SG and SC on the myocardial infarction of zebrafish. The blood flow software in Viewpoint ZebraBox was used to capture the aorta of zebrafish, and Zebra Blood (v3.4.2) analysis software was used to analyze the blood flow velocity of the aorta and cardiac output (CO). Compared with the normal group, the blood flow velocity was significantly reduced in the model group. Compound Danshen Dropping Pills (CDDP), all concentrations of SG and 1.95 μg/mL SC significantly increased the blood flow velocity of zebrafish in isoproterenol hydrochloride-induced zebrafish (Fig 6A). The cardiac output was remarkably reduced in isopropyl-adrenal hydrochloride induced in zebrafish, CDDP and SG can significantly increase the cardiac output, and SC had no significant effects on cardiac output (Fig 6B). The degrees of myocardial apoptosis were significantly increased in isoproterenol hydrochloride induced zebrafish, CDDP, 0.977 μg/mL SG, and 3.91μg/mL SC significantly reduced the myocardial apoptosis (Fig 6C and 6D). The myocardial infarction was also observed, and the myocardial cell area was significantly decreased in model zebrafish. CDDP, all concentrations of SG and SC significantly increased the myocardial cell area and mitigated myocardial infarction in isoproterenol hydrochloride-induced zebrafish (Fig 6E and 6F).

The myocardial muscle troponin T type 2α (tnnt2α) is a subtype of the myocardial troponin T gene in zebrafish. Tnnt2α mutations are associated with multiple types of cardiomuopathy [47]. Studies demonstrated that heart diseases such as dilated cardiomyopathy, acute heart failure, and congestive heart failure are associated with increased and decreased levels of the natriuretic peptide (NP) family, which have effective diuretic and natriuretic effects, as well as blood pressure lowering properties, and are peptide hormones that maintain cardiorenal homeostasis [48]. Atrial natriuretic peptide (ANP) and B-type or brain natriuretic peptide (BNP) are members of the NP family, which serve as translation biomarkers for the clinical treatment of heart failure [48, 49].

The expression of tnnt2α, ANP, and BNP increases when myocardial cell damage is the manifestation of diseases, such as acute myocardial infarction, coronary heart disease, and heart failure [47, 50]. In the model group, the expression of tnnt2α, ANP, and BNP significantly increased, then CDDP decreased the expression of tnnt2α, ANP, and BNP (Fig 6G–6I), and SG and SC did not affect the expression of ANP. 1.95 μg/mL SG and 3.91 μg/mL SC decreased the expression of tnnt2α and BNP in isoproterenol hydrochloride induced zebrafish. Meanwhile, 3.91 μg/mL SC was better effect compared with the same concentration SG. These results suggest the cardioprotective effect of SG was better than SC.

### Inhibitory effects of SG and SC on the inflammatory response of myocardial cells in isoproterenol-induced zebrafish

**Fig 7:**
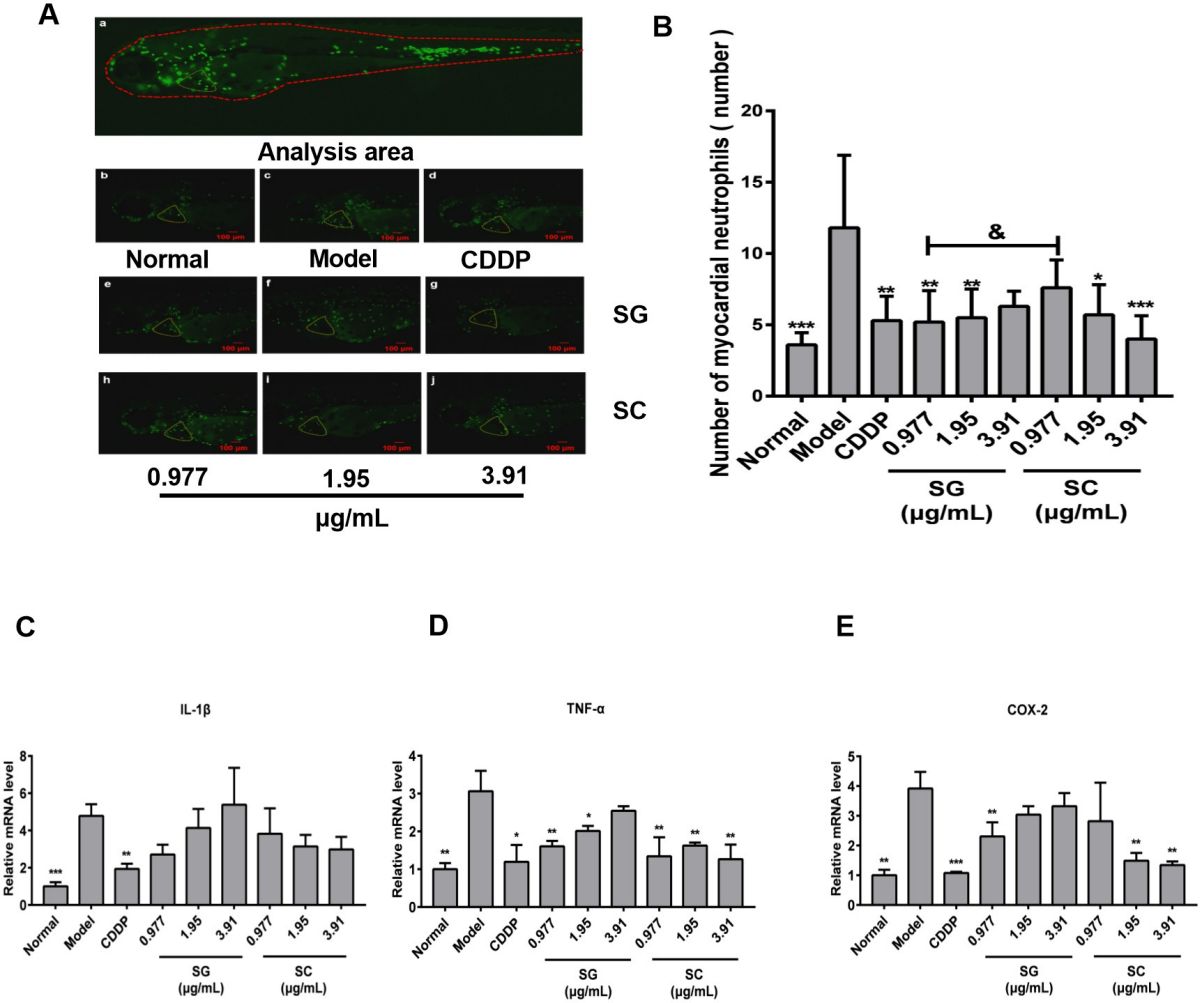
Anti-inflammatory effects of SG and SC. (A-B) Phenotypes of fish of Tg (MPO: EGFP) lines. (C-E) qRT-PCR analysis was performed to measure the inflammation-related genes IL-1β, TNF-α and COX-2 in Tg (MPO: EGFP) zebrafish. Images were captured at 400× magnification. Scale bars: 100 μm. The results are represented as a mean ± SE (n = 10). * *P* < 0.05, ** *P* < 0.01, *** *P* < 0.001 vs. model groups. ^&^ *P* < 0.05, vs.SC.

Myocardial infarction triggers an intense inflammatory response that is essential for myocardial repair but is also implicated in the pathogenesis of post-myocardial infarction remodeling and heart failure [51]. Myocardial injury is associated with inflammation and coagulopathy [52]. Neutrophils are a key component of the innate immune system. In heart injury, the retention of neutrophils can prolong the inflammatory period, leading to delayed scar regression and reduced cardiomyocyte proliferation [53]. Therefore, blocking the reaction of neutrophils is crucial to the repair of heart injury. The number of neutrophils was increased in isoproterenol hydrochloride-induced zebrafish (Fig 7A and 7B), while CDDP, the concentrations of SG (except 3.91 μg/mL), and the concentrations of SC (except 0.977 μg/mL) significantly decreased the number of neutrophils in model zebrafish (Fig 7A and 7B).

Furthermore, the effects of SG and SC on the expression of inflammation-related genes of myocardial in zebrafish were investigated including interleukin-1β, TNF-α, and cyclooxygenase-2 (COX-2). As shown in Fig 7, the mRNA expression of IL-1β, TNF-α, and COX-2 increased in isoproterenol hydrochloride-induced zebrafish (Fig 7C–7E). CDDP inhibited the expression of IL-1β, TNF-α, and COX-2 in model zebrafish, SG and SC suppressed the expression of TNF-α and had no effects on the expression of IL-1β, 0.977 μg/mL SG and 1.95–3.91 μg/mL SC significantly decreased the levels of COX-2 (Fig 7E) in isoproterenol hydrochloride-treated zebrafish.

Some studies have revealed the involvement of inflammation in myocardial damage in patients with sepsis [54]. The stimulation of many pro-inflammatory cytokines, such as IL-1β, IL-6, and TNF-α, may trigger several immune cascades [55, 56]. Heat shock protein 22 (Hsp22) suppressed the expression of inflammatory cytokines (IL-1β, IL-6, TNF-α, and NLRP3), and then ameliorated lipopolysaccharide-induced myocardial injury [54]. SG significantly decreased the levels of TNF-α and COX-2 in the isoproterenol hydrochloride-induced myocardial injury zebrafish, suggesting that SG served anti-inflammation and cardioprotective effects. IL-1β, activated by the NLRP3 inflammasome, compromises myocardial function. Pro-inflammatory cytokines IL-1β and TNF-α are significantly increased in both sepsis-induced cardiomyopathy patients and animal models [57]. Activation of COX-2 is a key target for chronic inflammation. When IL-17 binds to TNF-a, it may lead to inflammation by affecting COX-2 expression and PGE2 production. Reducing the level of COX-2, TNF-α, and IL-1β contributes to anti-inflammatory [36, 58]. These results indicate that SG has the same protective effect on myocardial inflammation as SC.

Collectively, SG is similar to SC in chemical composition, toxicological properties, antithrombotic activity, and myocardial infarction protection effects, and may be used as a substitute for styrax to reduce the collection from wild *L*. *orientalis* Mill. and increase the available styrax resources. Certainly, some limitation and further research direction should be discussed. Firstly, the comparison of toxicological and pharmacological properties between SG and SC should be conducted in rat, mice and even in humans. Secondly, the quantitative and qualitative dynamic change pattern of balsam secreted from the grafted *L*. *orientalis* Mill should be further investigated so as to effectively utilize grafted styrax resources. Thirdly, the effective chemical constituents for various pharmacological activities in SG and SC remains unclear, and should be further explored. Finally, the quality standards should be improved to effectively control the quality of styrax, and also identify the counterfeit and adulterate by rational use of new technical methods such as fingerprint and biotechnology.

## Conclusion

Styrax is an important ingredient of traditional Chinese medicine that showed definite and promising curative effects in the treatment of cardio-cerebrovascular disease. However, *L*. *orientalis* Mill. has been recorded in the Red List of Threatened Species as an endangered plant, and the Turkish government also banned the commercial collection of *L*. *orientalis* Mill. in the 1980s. The poor adaptability of *L*. *orientalis* Mill. in China results in the shortage of styrax, and then insufficient medical application. Grafting has the advantage of enhancing the adaptability of plants. We successfully grafted *L*. *orientalis* Mill. on *L*. *formosana* Hance and obtained styrax. The present study found that SG had similar to SC in chemical components, and toxicological and pharmacological properties. We conclude that SG can substitute SC, and be used in TCM preparation. Finally, this study may provide a promising new resource for styrax, and promote the sustainable application of TCM-containing styrax in clinical practice.

## Supporting information

S1 Table The chemical components of SG, SC, and SS. A total of 59 chemical components in styrax were identified by GC-MS.
(XLSX)
